# Minimally invasive and conventional surgical treatment of primary benign cardiac tumors

**DOI:** 10.1186/s13019-019-0890-2

**Published:** 2019-04-11

**Authors:** Congcong Luo, Jiaquan Zhu, Chunrong Bao, Fangbao Ding, Ju Mei

**Affiliations:** Department of Cardiothoracic Surgery, Xinhua Hospital, Shanghai Jiaotong University School of Medicine, 1665 Kongjiang Road, Shanghai, 200092 China

**Keywords:** Primary cardiac tumors, Myxoma, Minimally invasive surgery

## Abstract

**Background:**

Primary cardiac tumors are rare and the majorities are benign. Conventional surgical treatment uses median sternotomy, while minimally invasive surgery from right anterolateral minithoracotomy has become an alternative method in recent years. In this study, we summarized the surgical outcomes of both approaches.

**Methods:**

From January 2008 to August 2018, 50 patients with primary benign cardiac tumors underwent either conventional or minimally invasive surgery in our department. The baseline data were collected. The peri-operative data and follow up results were compared between the two groups.

**Results:**

There were19 men and 31 women enrolled in this study with a mean age of 55.0 ± 17.5 years. The most common site of the tumor was left atrium (*n* = 40, 80%), followed by right atrium (*n* = 8, 16.0%), right ventricle (*n* = 1, 2.0%) and left ventricle (n = 1, 2.0%). All patients underwent surgery uneventfully, including 33 cases (66.0%) of median sternotomy and 17 cases (34.0%) of right anterolateral minithoracotomy. No significant differences were found between the two groups in terms of cardiopulmonary bypass time, aortic cross-clamp time, postoperative intubation time, intensive care unit days and length of the hospital stay. Patients with right anterolateral minithoracotomy had less post-operative chest drainage (536 ± 159 vs 773 ± 255 ml, *P* < 0.01) and transfusion rate (5.9% vs 33.3%, *P* = 0.033) than those who had sternotomy. There was no peri-operative death, and all the patients were alive and free of recurrence at the latest follow-up.

**Conclusions:**

Surgical resection of primary benign cardiac tumors is safe, effective and durable. The right anterolateral minithoracotomy provides the same postoperative recovery as standard median sternotomy, but less transfusion. It can be considered as a promising alternative approach.

## Background

Primary cardiac tumor is a rare disease with an incidence ranging from 0.001 to 0.3%, and 75% of cardiac tumors are benign [1, 2]. Atrial myxoma is the commonest type [3–5], which accounts for more than half of the benign cardiac tumors. Echocardiography is the most valuable diagnostic imaging technique [6]. Surgical resection is the main treatment of cardiac myxoma and should be performed as early as possible when diagnosis is established because of risks of embolization and valvular obstruction [7–9]. The prognosis of cardiac myxoma is favorable after complete resection. Median sternotomy is the conventional approach for resection of cardiac tumors, but it accompanies with unsatisfied cosmetic outcome, risk of sternal infection and other possible complications. With the advancements of minimally invasive techniques in valve surgery, right anterolateral minithoracotomy incision is now gradually applied in the surgery of cardiac tumors; however, there are very few reports with limited cases [10–12]. In this retrospective study, we summarized our own experience using these two surgical incisions and aimed to provide alternative options for the treatment of cardiac tumors.

## Methods

This retrospective study was approved by our institutional research ethics board, and informed consent was waived. From January 2008 to August 2018, surgical patients with diagnosis of a cardiac mass in our hospital were reviewed and those who were confirmed benign tumors postoperatively were included in this study. We only included benign tumors for analysis and all malignant tumors were excluded because surgical resection for cardiac malignant tumor is still controversial, and the outcomes are quite different from benign tumors. In total, 50 consecutive patients were finally included in this study. Medical records of all these patients were reviewed, and data regarding preoperative evaluation, surgery, postoperative complication, and postoperative echocardiography were collected. In the earlier era of this study, a conventional median sternotomy (CMS) was used, while a right anterolateral minithoracotomy (RAMT) approach has been used in more recent years in patients who received elective surgery.

### Surgical techniques

1. Right anterolateral minithoracotomy group (RAMT group)

- A double-lumen endotracheal tube was inserted for unilateral ventilation during the surgery. Then patient was placed in supine position and right chest was elevated at an angle of 30°. The intercostal incision was estimated according to the preoperative chest computed tomography (CT) scan. Generally, the thoracotomy of RAMT was undertaken at a length of 6 cm in the fourth intercostal space between the anterior line and the mid-clavicular line. In young female patients, a right sub-mammary skin incision was made to avoid injury of mammary gland, then subcutaneous fat and mammary gland tissue were dissected from the fascia upward to expose the fourth rib. After unilateral lung ventilation was started, the pericardium was opened vertically 2 cm anterior to right phrenic nerve, and suspended to achieve adequate exposure. A 3-cm groin incision was made, and the femoral artery and vein were mobilized. After heparin was given, aorta cannula was placed in the femoral artery, and the inferior vena cava was cannulated through the femoral vein. The superior vena cava was cannulated percutaneously through right jugular vein with a 16 Fr cannula. The cardioplegia needle was inserted into the aortic root from the thoracic incision, and cardiopulmonary bypass was performed with mild hypothermia. Cold blood cardioplegia was given after the ascending aorta was clamped with a Chitwood clamp through the 4th intercostal space. After the heart was arrested, we incised the right atrium and atrial septum, then removed the tumor completely. Cold saline was used to rinse the cardiac chambers, and careful inspection was undertaken to ensure complete resection. The atrial septum was repaired by direct running suture or autologous pericardium patch. The aortic cross clamp was removed, and the heart was re-perfused. The right atriotomy was closed using double-layer continuous running sutures.

2. Conventional median sternotomy group (CMS group)

- The patient was placed in supine position and anesthetized. Then the surgery was performed via median sternotomy. Cardiopulmonary bypass was established using aortic bicaval cannulation, and mild hypothermia was used. Antegrade hyperkalemic cardioplegia was given to achieve cardiac arrest. Right atriotomy was used to remove the tumor in the right-side chambers, while additional atrial septostomy was performed in those who had the mass in left atrium or ventricle. Intra-operative transesophageal echocardiography was performed to access tumor residues, valve competency, residual interatrial shunt and cardiac function.

### Perioperative management and follow up

The postoperative patients were recovered in intensive care unit, and transferred to the ward when stable. The post-operative recovery courses and complications were documented. Pre-discharged echocardiography was performed routinely, and the patients were followed up annually.

### Statistical analysis

Categorical variables were expressed as frequencies and percentages, and were compared between the two groups using χ2 test or Fisher exact test. Continuous variables were presented as means ± standard deviations and compared using Student t-test or Mann-Whitney U test as appropriate. STATA 14.0 software (STATA Corporation, College Station, TX) was used for statistical analyses. A two-tailed *P*-value < 0.05 was considered statistically significant.

## Results

### Preoperative clinical data

Table 1 demonstrates the preoperative clinical data. There were 31 (62.0%) females, and the mean age was 55.0 ± 17.5 years. The main symptoms included palpitation, shortness of breath, exercise intolerance, transient syncope, and cerebral embolism. There were 3 cases of pleural effusion, including one left, one right and the other one bilateral pleural effusion. Preoperative cardiac arrhythmia was found in 7 cases, including atrial fibrillation (*n* = 4), premature atrial contractions (*n* = 2) and premature ventricular contraction (*n* = 1). There were 10 cases of pre-operative stroke, among which 5 patients presented cerebral infarction as initial symptoms. Hypertension and diabetes were the two major associated non-cardiac diseases.

**Table 1:** Preoperative clinical data

Characteristic	CMS group(*n* = 33)	RAMT group(*n* = 17)	*P*-value
Age(y)	54.45 ± 17.30	55.94 ± 18.24	0.752
Sex (female)	19 (57.6%)	12 (70.6%)	0.369
Weight (kg)	60.4 ± 15.7	54.1 ± 19.5	0.668
Patient history			0.024
Acute (history <1w)	3 (9.1%)	6 (35.3%)	
Subacute (1w – 1 m)	6 (18.2%)	0 (0.0%)	
Chronic (history > 1 m)	24 (72.7%)	11 (64.7%)	
Arrhythmia	4 (12.1%)	3 (17.6%)	0.677
Preoperative stroke	5 (15.2%)	5 (29.4%)	0.239
Fever	2 (6.1%)	0 (0.0%)	0.542
Leukocyte increase	1 (3.0%)	1 (5.9%)	1.000
Non-cardiac disease			0.320
Hypertension	4 (12.1%)	4 (23.5%)	
Hypertension and diabetes	3 (9.1%)	0 (0.0%)	
Other diseases	2 (6.1%)	0 (0.0%)	
Tumor site			0.544
Left Atrium	26 (78.8%)	14 (82.4%)	
Right Atrium	5 (15.2%)	3 (17.6%)	
Left Ventricle	1 (23.0%)	0 (0.0%)	
Right Ventricle	1 (3.0%)	0 (0.0%)	
Aortic insufficiency			
Mild	2 (6.1%)	1 (5.9%)	0.980
Mitral insufficiency			
Mild	8 (24.2%)	5 (29.4%)	0.693
Tricuspid insufficiency			0.197
Mild	8 (24.2%)	9 (52.9%)	
Moderate	3 (9.1%)	1 (5.9%)	
Severe	2 (6.1%)	0 (0.0%)	
EF(%)	61.1 ± 12.4	66.3 ± 4.6	0.386
FS(%)	33.2 ± 7.3	36.6 ± 3.7	0.550
LVEDD (mm)	45.5 ± 12.9	45.0 ± 6.8	0.319
LVESD (mm)	29.9 ± 8.9	27.6 ± 4.9	0.402
Estimated pulmonary arterial systolic pressure (mmHg)	23.4 ± 10.4	23.9 ± 6.2	0.586

*EF* ejection fraction, *FS* fractional shortening, *LVEDD* left ventricular end diastolic diameter, *LVESD* left ventricular end systolic diameter

Preoperative echocardiography showed that the tumor was most likely located in left atrium (*n* = 40, 80.0%), followed by right atrium (*n* = 8, 16.0%), right ventricle (*n* = 1, 2.0%), and left ventricle (n = 1, 2.0%). Among the 48 patients with mass in left and right atrium, 46 cases had obvious pedicles around the site of oval fossa, and the remaining 2 patients had pedicles located in the orifice of pulmonary veins (n = 1, 2.0%) and coronary sinus (n = 1, 2.0%). The tumor size measured by echocardiography was roughly estimated using length and width (cm). The tumor size ranged from 0.62 cm × 0.89 cm to 7.7 cm × 6.8 cm. The primary associated valvular disease was valvular insufficiency, included aortic insufficiency (*n* = 3), mitral insufficiency (*n* = 13), and tricuspid insufficiency (*n* = 23); while the valvular insufficiency was mild in most of these patients. Preoperative mean ejection fraction (EF) of left ventricle was 62.9 ± 10.6% and mean fraction of shortening (FS) was 34.4 ± 6.5%. According to the classification of New York Heart Association (NYHA), cardiac function of most patients was in grade II (*n* = 24, 48.0%), followed by grade III (*n* = 13, 26.0%), grade I (*n* = 12, 24%), and grade IV (n = 1, 2.0%). There was significant correlation between tumor size and NYHA heart function (the correlation coefficient was 0.628, *P* < 0.001). There was no significant difference between RAMT and CMS groups.

### Surgical outcomes of RAMT and CMS groups

All the 50 patients underwent surgery, including 33 cases (66.0%) of CMS and 17 cases (34.0%) of RAMT. There was no perioperative death or re-exploration for hemostasis. In CMS group, simultaneous procedure included mitral valvuloplasty in 3 cases, mitral valve replacement in 1 case, tricuspid valvuloplasty in 3 cases, and coronary artery bypass graft in 1 case. In RAMT group, one concomitant tricuspid valvuloplasty was performed. No one in RAMT group required transition to CMS during surgery. Intra-operative transesophageal echocardiography showed that there were no more than moderate residual valve regurgitations. One case had renal dysfunction after operation, and it was improved after medical treatment. Postoperative arrhythmia, mainly transient atrial fibrillation, was found in 11 patients in CMS group and 4 patients in RAMT group. Two patients had wound infection in CMS group and underwent multiple debridement with vacuum sealing drainage. There was no significant difference in aortic cross clamp time and cardiopulmonary bypass time between the two groups. The duration of intensive care unit (ICU) stay, postoperative intubation time, and duration of postoperative hospital stay were shorter in RAMT group, but the differences were not statistically significant. Patients with right anterolateral minithoracotomy had less postoperative chest drainage (536 ± 159 vs 773 ± 255 ml, *P* < 0.001) and transfusion rate (5.9% vs 33.3%, *P* = 0.033) compared to those who had sternotomy (Table 2).

**Table 2:** Comparison of postoperative recovery related indicators

Variables	RAMT (n = 17)	CMS (n = 33)	*P*-value
Aorta cross-clamp time (min)	30.2 ± 19.3	31.9 ± 22.5	0.715
Cardiopulmonary bypass time (min)	71.9 ± 21.4	64.8 ± 27.4	0.667
Intensive care unit days	2.6 ± 1.3	3.1 ± 1.7	0.216
Intubation hours	17.4 ± 11.2	19.1 ± 20.6	0.408
Postoperative hospital days	15.3 ± 4.3	18.0 ± 12.1	0.08
Postoperative complications			
Renal insufficiency	0	1	0.340
Atrial fibrillation	4	11	0.474
Wound infection	0	2	0.542
Postoperative chest drainage (ml)	536 ± 159	773 ± 255	< 0.001
Transfusion rate	1 (5.9%)	11 (33.3%)	0.033

*RAMT* right anterolateral minithoracotomy, *CMS* conventional media sternotomy

### Pathological findings

The pathological findings of the resected cardiac mass were summarized in Table 3. The main pathological type was myxoma, which was more often in women than men (30 vs 16, *P* = 0.127). Other pathological types included lipoma, rhabdomyoma and fibroma. The relationship between tumor pathology and location was showed in Table 3. Those masses within the left atrium in 40 patients were all found myxomas. Eight masses in the right atrium were found 5 cases of myxomas, 1 case of lipoma, 1 case of rhabdomyoma and 1 case of fibroma. There were 2 cases with masses in the ventricles, and they were lipoma and myxoma respectively. Myxoma often occurred in left atrium, while right atrium is more prone to grow non-mucinous tumors compared to left atrium(P<0.01).

**Table 3:** Histopathology of primary cardiac tumors

Pathology	No.	Sex		Tumor location			
		Male	Female	Left Atrium	Right Atrium	Left Ventricle	Right Ventricle
Myxoma	46 (92.0%)	16	30	40	5	0	1
Lipomas	2 (4.0%)	2	0	0	1	1	0
Rhabdomyoma	1 (2.0%)	0	1	0	1	0	0
Fibromas	1 (2.0%)	1	0	0	1	0	0
Total	50 (100.0%)	19	31	40	8	1	1

### Follow-up results

The mean postoperative follow-up was 41.4 ± 35.2 months (range 1–127.1 months). The patients recovered well during the follow-up period. No death, recurrence of tumor or reoperation occurred during the follow-up period. However, 3 patients in this study underwent previous operations of cardiac tumor in other hospitals 3, 4 and 16 years ago, and they were operated in our hospital for tumor recurrence.

## Discussion

### Characteristics of cardiac tumors

Primary cardiac tumors are rare, and the reported incidence ranges from 0.001 to 0.3% in autopsy studies [1, 2]. Primary cardiac tumors are mostly benign, and mainly located in the atria especially left atrium. The commonest primary cardiac tumor is myxoma. Cardiac myxomas are more common in women than men, with a 2:1 female preponderance [13]. Non-myxomatous neoplasms are prone to grow in right atrium, and include rhabdomyosarcoma, lipoma, capillary hemangioma, leiomyoma, fibroma, etc. It has been also reported that right atrium is more prone to grow malignant tumors than left atrium [14]. If a right atrial tumor is detected by echocardiography, it should be aware of the possibility of malignant tumor and cardiac magnetic resonance imaging (MRI) examination should be performed if necessary [15]. Our study found similar results with the abovementioned literatures. Eighty percent patients in this study had tumors in the left atrium, and all of them were myxomas; while 16.0% cardiac tumors were located in the right atrium with 5 case of myxomas and 3 cases of non-myxomatous neoplasms (lipoma, rhabdomyoma and fibroma respectively).

### Diagnosis of primary cardiac tumor

The symptom of primary cardiac tumor lacks of specificity, therefore early diagnosis is difficult. Preoperative symptoms include palpitations, dyspnea, embolism, and neurologic deficits, and they were closely related to tumor site and features. At present, transthoracic echocardiography is the primary imaging technique for diagnosis, and the detection rate is 95.2% [6, 16, 17]. It provides important information, such as tumor site, size, number, pedicle and hemodynamic changes, as well as ventricular function and valvular function. CT scan and MRI can provide relation between cardiac tumor and adjacent intracardiac/extracardiac structures, and may help differential diagnosis [13, 18]. Some patients were asymptomatic at early stage. In this group, 7 cases (14.0%) were founded cardiac tumor incidentally in physical examination. Most patients were diagnosed from clinical symptoms caused by valvular obstruction due to the cardiac tumor. Patients with symptoms of cerebral embolism should be alert of heart tumors and routinely undergo echocardiography to avoid missed diagnosis, and in this study, up to 20% patients had preoperative stroke.

### Surgical choices

Elective or even emergent surgery is the primary treatment of cardiac tumors. During induction of anesthesia, the vital signs should be carefully monitored in case of hypotension or cardiac arrest due to complete occlusion of tumor in heart valve. Conventional median sternotomy is widely used in primary cardiac tumors resection. This approach has excellent exposure; however, it also accompanies with some disadvantages, such as instability of the thoracic cavity, potential postoperative mediastinitis, and poor cosmetic effect [9]. Various minimally invasive surgical approaches have been applied in the operation of mitral and tricuspid valves in recent years, such as right thoracolumbar incision [19, 20], right axillary straight incision [21], and partial sternal incision [22]. Since Ko et al. reported the surgery of myxoma resection through right anterolateral minithoracotomy approach two decades ago [23], minimally invasive surgical techniques have been gradually applied to the treatment of cardiac tumors. However, Due to the rarity of primary cardiac tumors, such reports are limited. In addition, because of limited surgical exposure of minimally invasive approach, there are concerns of incomplete tumor resection. The safety and clinical efficacy of this operation are still controversial. Therefore, we summarized and compared clinical data between these two approaches. No patients using RAMT approach required intraoperative transition to CMS approach, and intraoperative echocardiograms proved complete resection in all patients of RAMT group. Patients in both groups recovered well and were discharged uneventfully without perioperative death. This suggests that RAMT approach in cardiac tumor patients is safe. Our study showed that the cardiopulmonary bypass time in the minimally invasive group is a little longer, and the aortic cross clamp time was similar between two groups, but these differences were not statistically significant. This might due to more concomitant procedures in CMS groups, which prolonged cross clamp time despite of the advantage of better surgical exposure. Postoperative intubation time and ICU stay in RAMT group were shorter than CMS group, but again there were no statistically significant differences. From our study, RAMT had the advantage of less postoperative drainage and less transfusion. Minimally invasive incision has been reported advantages of less trauma, shorter hospitalization time, and no risk sternal infection [24]. Among the 50 patients in this study, 2 cases in CMS group developed mediastinal infection which increased cost and hospital stay. Postoperative transient arrhythmia occurred in 11 patients of CMS group and 4 patients of RAMT group, but there was no statistically significant difference.

Video assisted and totally thoracoscopic surgical resection of left atrial myxoma had been reported in recent years [25–27]. These literatures proved the feasibility of thoracoscopic surgery in such disease, and yielded excellent outcomes. From these studies, the average cross clamp time was around 50 min and cardiopulmonary bypass time was around 120 min, which are acceptable, but still longer than the results of sternotomy or minithoracotomy. We also started using video assisted technique in very recent years, and one patient in this study underwent video assisted surgery uneventfully, and her incision was only around 4 cm.

Despite minimally invasive surgery has been gradually applied in resection of primary cardiac tumors in recent years, there is no standard criteria of using this approach [11]. Usually, dense pleural adhesion, previous right thoracotomy, peripheral vascular diseases, or severely decreased lung function precludes the use of minimally invasive surgery in such patients. Furthermore, based on our own experience, minimally invasive surgery is not recommended if the tumor diameter is too large (> 5 cm). We had one 78 years old female with a mass of 7 cm in the left atrium in this study, and a RAMT approach was used. During the operation, the mass was resected from the atrial septum uneventfully, but we were not able to take it out from the septal incision, and finally a bi-atrial perpendicular incision was made to get it out of the heart. Although we finished the surgery uneventfully, we learned lessons from this case that a giant mass in left atrium might cause hemodynamic instability and take longer cross clamp time using RAMT approach. In addition, RAMT approach is not recommended if a metastatic or malignant tumor is highly suspected, unless the tumor is isolated and less than 3 cm in diameter, which is expected to be completely resected. Furthermore, cardiac tumors arising from ventricle or atypically within the left atrium might not be suitable to use RAMT, and sometimes transaortic video assisted inspection was required in patients with tumor in left ventricle [28]. Generally, in practice, we tend to choose patients with stable hemodynamics and nearly normal heart function to perform minimally invasive surgery. If patients are unstable, CMS approach should be the first choice, and the cardiopulmonary bypass time should be minimized as much as possible to facilitate postoperative recovery. Finally, in those who underwent previous sternotomy, we prefer redo-sternotomy in patients with low predicted risk of redo-sternotomy, such as the three redo operations in this study; however, RAMT in redo cardiac operations is feasible based on our previous experience [29] and other literatures [19], and it is an alternative in those who have high risk of redo-sternotomy.

### Prognosis

The prognosis of primary benign cardiac tumors is well as long as completely tumor resection is achieved. Surgical outcome for cardiac myxoma is generally good, with 20-year survival rate of 85%, and recurrence rate after resection of approximately 5% in all patients with an initial diagnosis of atrial myxoma [13]. It is very important to completely remove the cardiac tumor and the atrial septal tissue near the tumor pedicles for preventing recurrence. Although no recurrence was recorded during the postoperative follow-up of the patients in our study, three patients in this group underwent second operations due to recurrence. All of the 3 patients had received myxoma resection previously in other hospitals, and one of the patients relapsed 16 years after the first operation. Therefore, echocardiography should be performed regularly after surgery.

Rhabdomyoma and fibroma are primarily detected in children and adolescents. Surgical resection is indicated in patients presented symptoms of obstruction or arrythmia. Complete tumor resection is ideal; however, if this goal is not achievable, release of obstruction and restoration of ventricular function are the aims. Surgical mortality of rhabdomyoma was acceptable in a recent systemic review, and tumor recurrence rate was as low as 2.6% [30]. In addition, rhabdomyoma has the feature of spontaneous regression [14, 30], and those who underwent incomplete resection or treated conservatively also had good prognosis. The prognosis of fibroma is good after complete resection, and the reported recurrence rate is 1.6% [30]. Lipoma is usually found in adults. It can be an isolated mass in cardiac chambers or present as lipomatous hypertrophy, which affects interatrial septum [14]. Surgical resection is the main treatment in symptomatic patients and obtains excellent outcomes.

### Limitations

This study is limited by its retrospective nature with few cases. Only short and median follow up data are available. Due to the concern of losing too much information and the small discrepancy of baseline data between the two groups, propensity score matching was not used. Prospective randomized controlled study in selected cases with longer follow up may confirm the advantages of minimally invasive surgery in the resection of cardiac tumor in the future.

## Conclusions

Myxoma is the most common type of primary benign cardiac tumors, and more often in the left atrium. Surgical resection is the primary treatment with excellent outcomes. Complete resection is the key to prevent recurrence, and regular echocardiography should be followed up after operation. The RAMT approach has the same efficacy and safety as the CMS approach. Additionally, the right minithoracotomy has the advantages of less postoperative drainage, less transfusion, improved cosmetic results, and less hospital stay, which can be considered as a promising alternative to CMS approach.

## Abbreviations

CMS: conventional median sternotomy
RAMT: right anterolateral minithoracotomy
